# The deconstructed procedural description in robotic colorectal surgery

**DOI:** 10.1007/s11701-024-01907-9

**Published:** 2024-03-30

**Authors:** Kirsten Larkins, Ned Quirke, Hwa Ian Ong, Jade El Mohamed, Alexander Heriot, Satish Warrier, Helen Mohan

**Affiliations:** 1 Department of Cancer Surgery, Peter MacCallum Cancer Centre, Melbourne, VIC Australia; 2 International Medical Robotics Academy, North Melbourne, VIC Australia; 3 Department of Surgery, University of Melbourne, Melbourne, VIC Australia; 4 University College Dublin School of Medicine, Dublin, Ireland; 5 Department of Colorectal Surgery, Austin Health, Heidelberg, Australia; 6 Department of Colorectal Surgery, Alfred Health, Melbourne, VIC Australia

**Keywords:** Robotic surgery, Robotic surgical training, Curriculum design, Colorectal surgery, Surgical education

## Abstract

Increasing robotic surgical utilisation in colorectal surgery internationally has strengthened the need for standardised training. Deconstructed procedural descriptions identify components of an operation that can be integrated into proficiency-based progression training. This approach allows both access to skill level appropriate training opportunities and objective and comparable assessment. Robotic colorectal surgery has graded difficulty of operative procedures lending itself ideally to component training. Developing deconstructed procedural descriptions may assist in the structure and progression components in robotic colorectal surgical training. There is no currently published guide to procedural descriptions in robotic colorectal surgical or assessment of their training utility. This scoping review was conducted in June 2022 following the PRISMA-ScR guidelines to identify which robotic colorectal surgical procedures have available component-based procedural descriptions. Secondary aims were identifying the method of development of these descriptions and how they have been adapted in a training context. 20 published procedural descriptions were identified covering 8 robotic colorectal surgical procedures with anterior resection the most frequently described procedure. Five publications included descriptions of how the procedural description has been utilised for education and training. From these publications terminology relating to using deconstructed procedural descriptions in robotic colorectal surgical training is proposed. Development of deconstructed robotic colorectal procedural descriptions (DPDs) in an international context may assist in the development of a global curriculum of component operating competencies supported by objective metrics. This will allow for standardisation of robotic colorectal surgical training and supports a proficiency-based training approach.

**Supplementary Information:**

The online version contains supplementary material available at 10.1007/s11701-024-01907-9.

## Introduction

With increasing utilisation of robot surgical technology in colorectal surgery, training has become a focus of discussion internationally. Development of training curricula has focussed primarily on basic operational skills at the console and the bedside [1]. International consensus has renewed recommendations for standardisation of robotic colorectal training [2, 3]. Specialty specific training in colorectal surgery, however, is not yet standardised and has been implemented in differing formats internationally [4, 5]. Furthermore, defining standardised objective assessments for accreditation can be challenging, as discussed in a recent systematic review [6].

Although variation in training structure is inevitable, there is a common challenge of meeting proficiency standards to maintain patient safety while the novice surgeon navigates the learning curve [7]. Several published colorectal training curricula have therefore introduced component operating to give robotic surgical trainees graded autonomy through their training under guidance of a robotic surgical proctor [8]. This approach to integrating training allows for trainees to participate at a level appropriate for their level of robotic operating skill once they have completed their simulation-based basic robotic skills training [9].

Training by component operating relies on the ability to break down colorectal surgical procedures into a consistently reproducible operative approach. This process is often referred to as procedural deconstruction and has been proposed as a solution for challenges in modern surgical training including generating a meaningful record of trainee participation in operative cases [10]. Procedural deconstruction can be achieved through review of publications of procedures [11], generation of expert opinion [12], cognitive task analysis [13] or more recently though machine learning and artificial intelligence [14]. Educationally, procedural deconstruction has additional learning benefits. Having a procedural description can assist trainees in learning complex cases by finding common features in existing knowledge [15]. It has utility in reducing the mental effort required to learn a new task by breaking it down into more approachable chunks of knowledge [16, 17], and can improve performance of procedural skills [18, 19]. Procedural deconstruction has even be used to guide novel training approaches such as teaching appendicectomy to a non-surgically trained space team [20].

The result of deconstructing an operative procedure is often published as a “standardised procedure”. This can then be used to develop a specific assessment approach which is essential for a proficiency-based approach to training [21, 22]. In robotic surgery there is a growing importance of delivering training up to a specified proficiency standard and utilisation of a wider range of assessment metrics [23, 24]. Further development of these metrics in robotic surgical training would benefit from deconstructed procedural descriptions, allowing more targeted, procedure specific feedback for learners. Integration of novel technologies in training can then be structured around a component-based approach based on deconstructed procedural descriptions [25].

This systematic scoping review aims to identify which robotic colorectal operations have undergone procedural deconstruction and have published procedural descriptions. The literature will be used to develop an understanding of how these descriptions been determined and been incorporated into training. This information will be synthesised to present approach to educational design in robotic colorectal surgery using component operating and proficiency-based progression training.

## Methods

**Fig. 1:**
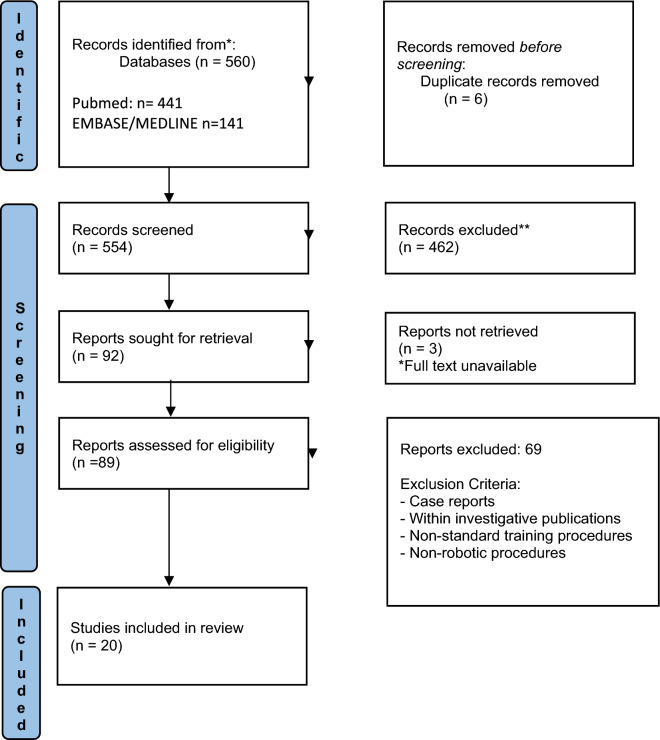
PRISMA flow diagram of included studies

A systematic literature search was conducted in June 2022. Databases included were PubMed, Embase and Medline. The search strategy included three groups of keyword terms combined with the Boolean operator AND (colorectal, colon, rectal), (procedure, standard, guide, steps, experience) and (robotic, surgery, minimally invasive). The full strategy is available as appendix a. Abstracts generated from this literature search were exported into literature management software for blinded review. Two reviewers (KL and NQ) independently assessed abstracts with papers extracted for full text review if they met the following inclusion criteria: papers describing a robotic colorectal procedure, published in English language, full text available published between 2002 and 2022. The relevant exclusion criteria were procedures described in the context of a case report or embedded within investigative publications and non-robotic or non-standard training procedures. The full PRISMA search is included in Fig. 1

Data were extracted for included publications with disagreements for inclusion resolved by a third reviewer (HM). Simple descriptive statistics were calculated for mean number of procedural components.

## Results

### Colorectal procedures included

Table 1 summarises the included studies. Procedural descriptions were retrieved for ten procedures across the 20 included articles. There was significant heterogeneity in the naming and description of the procedures performed. Procedural descriptions were identified for total mesorectal excision (*n* = 6) [26–31], abdominoperineal resection or proctectomy (*n* = 7) [32–38], left colonic and anterior resections (*n* = 5) [39–43], and reversal of Hartmann’s procedure (*n* =1) [44]. Described approaches to robotic colorectal operating components were also identified for port placement (*n* = 1) [45] and intracorporeal anastomosis (*n* = 1) [43].

**Table 1:** Details of included publications

Authors	Year	Procedure	Components
Procedures involving total mesorectal excision (TME)			
Hellan et al.	2009	Low anterior resection with TME and splenic flexure mobilization	1. Positioning of the patient 2. Port placement and positioning of the da Vinci robot 3. Mobilization of splenic flexure and left colon with high ligation of the IMA 4. TME with low division of rectum 5. Specimen extraction
deSouza et al.	2010	Total mesorectal excision	1. High ligation of the IMA and medial to lateral mobilisation of the descending colon using laparoscopy 2. IMV divided and splenic flexure mobilized where necessary using laparoscopy 3. Da Vinci S robot positioned between patients' legs and docked 4. Rectal mobilization and full TME 5. Robot undocked and anastomosis performed with using either double-stapled or double-purse string technique
Priatno et al.	2015	TME	1. Operating room set-up, patient positioning and ports placement 2. Abdominal phase: vascular ligation and sigmoid colon to splenic flexure mobilization 3. Pelvic dissection phase 4. Rectal reconstruction with or without ileostomy
Pesi et al.	2017	Low anterior resection	1. Patient positioning 2. Port placement 3. Identification, ligation and division of the inferior mesenteric vessels 4. Splenic flexure mobilization 5. TME 6. Rectal transection
Herrando et al.	2022	Low anterior resection	1. Ports placement and robot docking 2. Positioning and exposure 3. Vascular dissection and ligation 4. Colon mobilization in a medial to lateral fashion 5. Splenic flexure mobilization with 3-dimensional traction step 6. Mesorectal excision: dissection started from a posterior approach, followed by lateral approaches and ending with the posterior approach 7. Rectal section and colorectal anastomosis with previous indocyanine green test
Bae et al.	2015	Left colectomy/anterior resection	1. Installation and docking 2. Lymphovascular dissection and autonomic nerve preservation 3. Splenic flexure mobilization 4. Redocking 5. Rectal dissection and anastomosis
Abdominoperineal resection or proctectomy			
Park et al.	2010	Low anterior resection ± splenic flexure mobilization APR	Two phase - Lateral phase and pelvic phase 1. Port placement 2. Patient positioning 3. Procedures—lateral phase and pelvic phase lateral phase 1. Medial to lateral dissection 2. Ligation and division of IMA 3. ± Splenic flexure mobilization 4. Robot arms reconfigured to facilitate pelvic phase Pelvic Phase 1. Dissection of pelvic cavity 2. Division of mesorectum 3. Division of rectum with endo linear stapler 4. Laparoscopic anastomosis
Kang et al.	2011	APR	1. Robot and ports set-up 2. Patient's position and preparation 3. Lateral phase 4. Pelvic phase 5. Perineal phase 6. Making colostomy
Kang et al.	2012	Extralevator APR	1. Patient positioning and port placement 2. Laparoscopic sigmoid colon mobilization in medial to lateral fashion 3. Laparoscopic ligation of IMA 4. Laparoscopic mesenteric and colon division (robot docked) 5. TME (robot undocked) 6. Perineal incision made 7. Specimen delivered 8. Perineum closed
Bertrand et al.	2016	Proctectomy	1. Preconditioning and anaesthesiology 2. Patient's positioning 3. Port placement 4. Robot docking 5. Technique description 6. Conclusion
Tamhankar et al.	2016	Aim to describe technique for any rectal resection as part of: APR Intersphincteric resection Anterior resection	1. Port placement 2. Robot docking 3. Medial to lateral dissection 4. Splenic flexure mobilization 5. Total mesorectal excision 6. Rectal transection 7. Specimen extraction 8. Stapled or hand sewn anastomosis
Ahmed et al.	2016	Rectal resection in: Anterior resection APR Completion proctectomy Hartmann IPAA	1. Theatre setting and patient positioning 2. Port placement for left colonic and splenic mobilization/Port placement for pelvic dissection 3. Initial setting and exposure 4. Left colonic mobilization and vascular control 5. Lateral colonic and splenic flexure mobilization 6. TME 7. Anastomosis
Hollandsworth et al.	2020	Subtotal colectomy, total proctocolectomy	1. Patient positioning 2. Access and port placement 3. Caudal dissection—differentiates between procedure for subtotal colectomy and total proctocolectomy 4. Cephalad dissection 5. Extraction
Left colonic or anterior resection			
Miskovic et al.	2019	Anterior resection	1. Setup 2. Port positioning 3. Docking 4. Colonic mobilisation 5. Pelvic dissection 6. Specimen extraction and anastomosis
Tou et al.	2020	Low anterior resection	1. Patient positioning and preparation 2. Preparation of the operative field 3. Trocar position 4. Docking 5. IMA dissection/ligation 6. IMV exposure and ligation 7. Splenic flexure mobilization 8. Complete mobilization of the left colon 9. Rectal dissection/transection 10. Undocking system 11. Specimen extraction 12. Anastomosis 13. Stoma formation and wound closure 14. Transfer Patient to bed
Toh et al.	2020	Low anterior resection	1. Robot positioning and docking 2. Robotic dissection phase 1 3. Repositioning camera, arms and instruments 4. Robotic dissection phase 2 5. Stapling the rectum 4. Exteriorisation, resection and anastomosis
López et al.	2022	Left hemicolectomy with intracorporeal anastomosis	1. Preparation 2. Patient positioning 3. Port placement 4. Resection 5. Anastomosis 6. Specimen and trocars removed and sites sutured
Hollandsworth et al.	2022	Robotic left stapled total intracorporeal anastomosis	Describes access and port placement for: 1. Proctectomy 2. Sigmoidectomy Describes dissection for each operation briefly Describes Two techniques for ICA 1. Anvil forward 2. Anvil backward Describes a method for partially ECA
Other			
Giuliani et al.	2020	Reversal of Hartmann's	1. Patient positioning 2. Port placement 3. Adhesiolysis + mobilization of rectal stump 4. Splenic flexure takedown 5. Anastomosis
Lee et al.	2020	Only describes port placement 1. TME + L colectomy 2. R colectomy 3. Mesh ventral rectopexy 4. Trans anal approach	Describes port placement only

Across the 13 publications that described an approach to rectal resection there was a mean number of 7 procedural components (range 4–14). The included procedural descriptions described the following procedural components; set-up (including patient and port positioning and docking), dissection/mobilisation, vascular control, resection, reconstruction (anastomosis ± stoma formation).

### Determining procedural descriptions

Due to significant heterogeneity of the data presented, and limited data across a number of colorectal procedures, it was not appropriate to syntheses the available literature into a proposed deconstructed procedural description. The majority of the data describes robotic techniques relating to rectal dissection, however, lack a consistent structure and the lack of a methodologically rigorous approach to development of the procedural descriptions limits their utility in a training context. The most structured and applicable publications identified in this review [39, 40] have been used to guide the development of a proposed approach to the use of deconstructed procedural descriptions in training. These publications have been used as a model given their rigorous approach to development of the description, detail included in the description allowing for meaningful assessment, and inclusion of a defined objective of informing training.

Based on the evidence collated, the following definitions have been formulated following a process of expert opinion to guide educational design moving forward in robotic colorectal surgery (Table 2). A procedure is deconstructed into components, which are a unit of meaningful operative autonomy consisting of either a procedural phase or step [40]. The simplest arrangement of these components to complete the operation (for example the least number of changes in anatomical change in focus and logical progression) is defined as the training approach. This acknowledges that there are multiple possible combinations of phases and steps that can be used to safely complete the operation. A standardised component therefore refers only to components of the procedure that are required to be completed in a specific fashion for a quality, safety, or oncological outcome marker. A deconstructed procedural description is therefore the foundation of this approach to training where the procedure is deconstructed into components by identifying the operative phases and steps. This should be achieved through a process of review which may include cognitive task analysis, and expert consensus preferably using a Delphi methodology. Process and procedural errors can thus be identified and mapped to their respective procedural components. This allows the development of objective assessment metrics to support a proficiency-based progression training approach.

**Table 2:** Definitions^a^

	Definition	Relevance
Proficiency based progression training (PBP) (49)	An educational approach that utilises objective metrics to assess learner performance compared to pre-determined proficiency benchmarks	Provides overarching structure to the approach for training and assessment in robotic surgery
Deconstructed procedural description (DPD)	A description of an operation outlining procedural phases and steps that has been constructed using a process of review and expert consensus	A DPD is a recipe for an operation and encompasses alternative approaches in operative technique in a systematic fashion
Component	A unit of meaningful operative autonomy—either a complete procedural phase, or number/ combination of procedural steps	Components can be obsessively assessed, customisable for the trainee’s skill level and graded for task difficulty
Procedural phase (36)	A group or series of integrally related events of actions that when combined with other phases make up or constitute a complete operative procedure	A larger, more significant component of assessable operative autonomy
Procedural step (36)	A component task that series aggregate of which forms the completion of a specific procedure	A smaller component of operative autonomy that can be assessed
Error (36)	A deviation from optimal performance	Can be determined by component and used for assessment
Critical error (36)	A major deviation from optional performance which has a likelihood of causing harm to the patient or compromising the safe completion of the procedure	Can be determined by component and used for assessment
Training approach	Components arranged in an approach that represents the simplest progression through the operative procedure	Based on the least number of changes of view/ instrumentation, logical progression, and application of basic skills
Standardised component	Component deemed oncologically or procedurally significant and therefore to done only in the described fashion	Marker of quality and safety—for example TME

^a^Three expert robotic colorectal surgeons involved in robotic surgical education reviewed the available literature and participated in group discussion to formulate the above definitions

### Approach to educational design

Methods of development for each procedural description varied. No mention of a specified method of development was seen in 13 of the descriptions (65%). Expert opinion (procedure agreed upon by two or more surgeons) was cited by five articles [34, 35, 38, 43, 44]. Two articles described a formal consensus process using a Delphi methodology [39, 40].

### Application in training

Inclusion of how the procedure was or could be used in an educational context was seen in five articles. Miskovic et al. discussed the value of adopting standardised procedures for training in their discussion [39]. Tou et al. in their European consensus, sought to develop procedural phases for the purpose of developing a standardised colorectal surgery curriculum [40]. The learning curve involved in developing robotic surgery skills was referred to by both Toh [41] and Hollandsworth [38]. The video and article by Herrando et al. were intended for the purpose of training surgeons [30].

## Discussion

This review has identified the current scope of procedural deconstruction for the purpose of forming procedural descriptions and their role in directing educational design in robotic colorectal surgery. There are limited procedural descriptions across procedures commonly performed within a colorectal robotic surgical scope of practice. There are, however, published descriptions across most of the core set of robotic colorectal procedures, with the notable exclusion of rectopexy and right hemicolectomy. This is despite robotic right hemicolectomy being identified as a complete core training case in robotic surgery [46].

Currently four published robotic colorectal surgical training programs utilise component operating [4]. In this recent review by Harji et al. (2022) although component operating was included as part of the structure of robotic colorectal surgical training, a lack of detail of key progression criteria and consistency in proctoring approach were highlighted [4]. This could be addressed through adapting standardised procedural components based on consensus published procedural descriptions to a structured progression of component skill.

Component training has been shown to be safe and feasible even in complex procedures such as rectal dissection [8, 9]. Even between these two examples of parallel component-based training in robotic rectal surgery the approaches differ, with one training programme breaking down the procedure into 11 steps [8] and the other using five component parts [9]. Although not based on a procedural description this approach to rectal dissection has been easily implemented, improving access to robotic console operating and assisting in directed, objective goal setting for trainers and trainees in the proctoring phase. Adopting a common approach through the use of deconstructed procedural descriptions also strengthens data comparability, which could provide useful feedback to improve the programmes as a whole.

The role of this review was to collate published procedural descriptions to allow for the consolidation of these into assessable components to guide training in robotic colorectal surgery involving core procedures only; however, several limitations are noted with the current available data. Advanced or complex cases were excluded as they are not the focus of initial design of curriculum in robotic colorectal surgery. This, however, demonstrated the publication bias in publishing procedural descriptions as journals tend to publish innovation and advanced approaches in the form of case reports, series, or video vignettes. One-off, unique case reports do not represent a form of systematic development of knowledge or consensus opinion and are therefore not appropriate for generation of a training curriculum. Whilst important to support this avenue of academic surgical progress, equity should be given to training approaches as well as novel technologies in publishing. A dedicated international approach to a training library may assist with this. Similarly, it is acknowledged that there will be far more published descriptions of robotic colorectal surgical procedures within other publication types, such as interventional trials and systematic reviews and metanalyses. As a training tool however, these embedded descriptions have limited searchability and accessibility for trainees. Additionally, most of these procedural descriptions have a focus separate to training, limiting their application to training design.

From an educational perspective, incorporating procedural descriptions has benefits for both trainees and trainers. Structured guidance reduces the cognitive load of planning and facilitating from the trainers, leading to improved opportunity and autonomy for trainees. This also creates a shared language that trainers and trainees can use to structure an individualised training pathway based on individual skill and speed of skill acquisition.

Procedural deconstruction for the development of procedural descriptions and component-based operative training in robotic colorectal surgery becomes increasingly relevant when considering its potential applications. Firstly, international consensus for training represents an important step towards developing patient safety standards in robotic colorectal surgery [39], which is essential to progress towards internationally recognised accreditation in robotic colorectal surgery.

There are challenges in adopting component operating based on procedural descriptions. There is discussion as to whether standardisation of a procedure is the right approach to training in surgery. This stems from concern that standard approaches limit innovation and adaptability in trainees and teach static and fixed thinking in the operating room [47]. Similarly, there is surgeon-to-surgeon variability which cannot be captured with a standard approach [48]. This requires a change in terminology as recommended and a change in mindset of proctoring surgeons to teach first the training approach, giving trainees the building blocks to then develop their own approach, incorporating any standardised components as required.

It is noted that the methodology for generating consensus opinions is associated with a significant time and resource cost especially when considering the depth and breadth of robotic colorectal surgical procedures. An approach addressing this may be through distilling the curriculum to a set of core colorectal procedures with critical component competencies that when acquired, are transferable to performing other, non-core procedures.

At a system-based level, guidance is required regarding the appropriate integration of this approach. This requires assessment of the cognitive and technical difficulty of each procedural component and benchmarking to ensure appropriate graded progression. Assessment of the appropriate difficulty of procedural steps for a trainees may be achieved through learning curve analysis, with steps assigned to the component console surgeon in consideration of their previous experience. Further studies into the standardisation of assessment of technical skills is required [6].

## Conclusion

This review highlights the value in development of procedural descriptions and their utility in structured component-based robotic colorectal surgical training. Further dedicated effort should be directed towards development of deconstructed procedural descriptions (DPDs) to guide component-based parallel training in robotic colorectal surgery and build an international approach to robotics education. Future directions should be focused on enhancing the educational value of this approach. With improvements in technology this includes a proficiency-based progressive training approach which harnesses individualised data collection to guide trainees through component operating appropriate for their level of skill, and structured development of competency and proficiency standards for robotic colorectal operative components.

## Supplementary Information

Below is the link to the electronic supplementary material.

Supplementary file1 (DOCX 106 KB)

## Data Availability

The datasets generated during and/or analysed during the current study are available from the corresponding author on reasonable request.
