# Supplementary material for: The deconstructed procedural description in robotic colorectal surgery
Source: J Robot Surg. 2024 Mar 30;18(1):147. doi: 10.1007/s11701-024-01907-9 (PMC10981632; doi:10.1007/s11701-024-01907-9)
Supplement: Supplementary file 1 — Supplementary file1 (DOCX 106 KB) [file 11701_2024_1907_MOESM1_ESM.docx]

**Appendix A**: Search Strategy

**PUBMED**

Right Hemicolectomy OR colectomy OR rectal OR rectopexy OR TME OR Hartmann’s OR anterior resection AND Robot OR robotic AND Consensus OR standard OR standardised OR description OR procedure

"Right Hemicolectomy"[Title/Abstract] OR "colectomy"[Title/Abstract] OR "rectal"[Title/Abstract] OR "rectopexy"[Title/Abstract] OR "TME"[Title/Abstract] OR "Hartmann's"[Title/Abstract] OR "anterior resection"[Title/Abstract]) AND ("Robot"[Title] OR "robotic"[Title]) AND ("Consensus"[Title/Abstract] OR "standard"[Title/Abstract] OR "standardised"[Title/Abstract] OR "description"[Title/Abstract] OR "procedure"[Title/Abstract])

**OVID – MEDLINE and EMBASE**

**
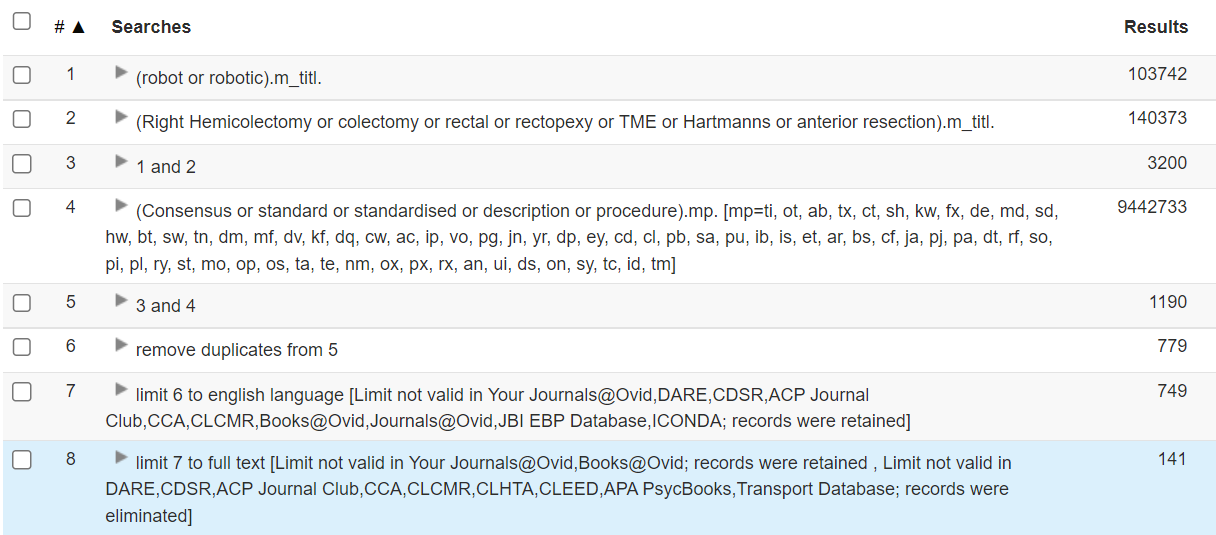
**
